# Role of Efficient Human Resource Management in Managing Diversified Organizations

**DOI:** 10.3389/fpsyg.2022.864043

**Published:** 2022-04-20

**Authors:** Huang Minghua

**Affiliations:** School of Economics and Management, University of Science and Technology of China, Hefei, China

**Keywords:** training and development, performance appraisal, diversity climate, job satisfaction, human resource

## Abstract

As the world has turned into a global village, it has created many challenges for human resource departments regarding the management of a diverse workforce in satisfying the employees and creating a diverse yet safe environment for them that does not make them uncomfortable. The current study has investigated the effect of human resource practices on the diversity climate with the mediation of job satisfaction. The data has been collected from human resource personnel of multinationals in China with the help of 316 participants. The study deployed SEM analysis to analyze and measure the effect of training and development along with performance appraisal on the diversity climate. The findings of the study revealed that training and growth or development do not have an impact on the diversity climate, however, performance appraisal has a strong positive impact. Similarly, the mediating role of job satisfaction has been found to ensure the relationship of training and development and performance appraisal with the diversity climate. This study has provided certain implications for the HR managers of multinationals to ensure a secure diversity climate for a diverse workforce.

## Introduction

As companies grow progressively or increasingly competitive, organizations have endured and continued to expand their goods and services to strive and compete more proficiently on a global scale; however, this can only be accomplished *via* the development of a highly qualified workforce. Businesses frequently have difficulty obtaining skilled workers. As a result, companies are compelled to look for talent in other nations, resulting in more cultural diversity in the workplace. Although cultural diversity has numerous dimensions, it is seen as an essential component of human resources and a perpetual challenge for enterprises to handle efficiently. Awais Bhatti et al. (2014) suggested that, if handled properly, cultural diversity may help firms acquire a competitive edge in both local and international markets. Furthermore, Shen et al. (2009) noted that while diversity concerns differ by nation, gender disparity and multicultural lineages are the most prevalent diversity challenges throughout the world, particularly in China. Multinational corporations all around the world have a cross-cultural and multicultural workforce (Shen et al., 2009). Without monitoring or regulating the job performance of a multicultural workforce, organizations will not be able to reach their desired goals.

Optimizing the job performance of something like a culturally diverse staff is a two-way process. To begin with, organizations with multiethnic or multicultural workforces should transition from domestic human resource management practices to global human resource management practices that support multicultural environments and foster psychological diversity climates to improve employees’ job satisfaction (Cletus et al., 2018). Cletus et al. (2018) claimed that local human resource management slants and methods are unable to deal with cultural variations that occur across ethnic origins by just instigating and applying diversity initiatives (Aluwi and Saihani, 2013). To develop a diverse environment, researchers have been attempting to emphasize which global human resource management performances and techniques are vital to boost job satisfaction and the performance of multicultural workforces (Bhatti et al., 2019). The way the world is inhabited and how we perceive the world is changing as a result of global diversity. Global diversity is visible; whether a corporation is global or not it affects us all, either explicitly or implicitly. Alliance or collaboration and cross-cultural teamwork are critical for an organization’s success.

People should learn to perceive their diversity as strengths rather than weaknesses if they have to function successfully. What matters more than the labels we use is what they signify. Managing diversity is a realistic business approach that focuses on optimizing the efficiency, innovation, and dedication of the workforce while satisfying the requirements of varied customer groups, even though it is built on cultural transformation. In basic words, diversity refers to “transcendence,” or human characteristics which are distinct from our own and exist outside of the groups to which we belong. There is a whole variety of characteristics that distinguish one person from another. According to Agrawal (2012), diversity is defined as “distinguishing oneself from others by possessing separate or dissimilar components or features.” This includes differences in age, culture, training, employment position, family structure, ethnicity, ethnic origin, personal features, gender, geographical heritage, religion, sexual preference, and working style in the workforce. Workers’ common opinions regarding a collection of human resource procedures geared at identifying and embracing differences between individuals are referred to as diversity climate.

The diversity environment, in particular, includes shared organizational perceptions about how devoted a company is to diversity-friendly management practices (e.g., diversity coaching, hiring, and mentoring) and diverse leadership that values all demographic groups (Choi, 2012; Opstrup and Villadsen, 2015). Since affirmative action alone is insufficient to allow the growth of a diverse workforce, public organizations additionally support management initiatives and expenditures that foster the absorption of individuals with diverse backgrounds to boost efficiency and competitiveness (Naff and Kellough, 2003). One of the most effective methods to help job seekers see a company as a great workplace is through diversity recruitment. Diversity training programs are frequently designed to reduce inter-organizational disputes and promote competitiveness in a diverse workforce. Moreover, by allowing the expression of original ideas and minimizing stereotyped attitudes, diversity-oriented leadership for the representation of all parts of society helps workers to fully contribute to organizational success (Gupta et al.). Employees acquire a common sense of a supportive diversity environment when a business is devoted to diversity-friendly managerial practices (e.g., diversity training, hiring, and mentorship) and diversity leadership to appreciate all demographic groups (Oberfield, 2016).

To manage personnel, human resource professionals have used many strategies such as recruiting and selection, training and development, performance review, and remuneration. Furthermore, as corporate settings evolve and the workforce becomes more diverse, the essence of these practices has shifted. As a result, while applying any method or strategy in managing workers, human resource professionals took into account the diversity factor to be more effective in managing a heterogeneous workforce. The human resource department deals with inequities in recruiting, training, performance appraisal, and incentives (Goodman et al., 2003; Kabongo et al., 2011). Managers have steadily improved the status of equitable employment chances and boosted innovation in multicultural workforces by applying human resource management toolkits. Training and development are important human resource tasks that assist firms in providing their staff with the necessary information, talents, and abilities. The conventional literature on training and development has established that training and development activities assist employees in gaining the essential skills and capacities to complete job tasks, as well as potentially improving overall job performance (Bhatti et al., 2019).

Traditional training and development processes, including recruiting and selection, may not be appropriate and beneficial for a multicultural workforce. Because multicultural employees differ in terms of personality, behavior, requirements, culture, and abilities, training and development activities may differ from those used in traditional workplaces (Bhatti et al., 2019). As a result, businesses should conduct a thorough assessment of their training needs and build training programs appropriately. Through this, Roberson et al. (2003) stated that when working within a diverse workplace, human resources professionals should do a thorough assessment of training needs and offer training programs that meet corporate objectives. Moreover, Kossek et al. (2006) noted that, while diversity training may cause short-term employee disagreement, the external trainer engaging in diversity training may assist in achieving greater productivity in the long run. Furthermore, Aldoghan et al. (2019) recommended that managers and collaborators should be conscious of their prejudices and engage in ongoing diversity training since solitary training sessions would not improve employee behavior.

Performance appraisal systems, to satisfy employees, and job performance have all been the subject of much research in the past (Awais Bhatti et al., 2014). Transparent and fair performance appraisal systems, according to previous research, assist firms in satisfying their employees, which leads to improved job performance. In this case, the point of contention is that a traditional assessment method may not be as effective or appropriate when working with such a diversified workplace. Management should create more customized performance assessment methods that can meet the demands of multicultural workforces in the climate of diversity. The performance evaluation committee, for example, should reflect all of the organization’s ethnicities, nationalities, and age groups (Xiaolong et al., 2021). In this context Kossek et al. (2006) advised that minorities be involved in the committee which evaluates, selects, and promotes individuals in order to properly execute the performance assessment method in a diverse workforce. Moreover, rather than focusing on personality, the assessment language should focus on performance (Huo et al., 2022).

Studies have looked into the link between job satisfaction and work performance in the past, but the findings have been conflicting, leaving the relationship between these two factors unclear. According to Yang and Hwang (2014), only a small amount of research has looked at the link between work happiness and job performance. Various individual elements which affect job satisfaction might explain the conflicting results. While previous research has shown that job happiness is an essential factor that impacts work performance, human resource professionals are still attempting to figure out what function job satisfaction plays in the success of multicultural workforces (Champathes Rodsutti and Swierczek, 2002). Nolan (2012) stated that globalization has significantly altered the aspects of work for employees, posing a tough opportunity for management staff seeking to improve employee job satisfaction.

According to Awais Bhatti et al. (2014), no previous research has previously focused on the importance of diverse climates in multicultural contexts. To fill this gap, this research might explain how diverse human resource strategies, such as cross-cultural training and employee performance assessment, can have an impact on diverse climates. They evaluated the performance of the faculty members in response to various human resource practices such as training and performance appraisal systems but could not evaluate the impact of human resource practices on modeling the diversity climate. Moreover, job satisfaction was suggested to be explored as a mediator between human resource practices and diverse climates. So, based on the analogy drawn in previous segments of the introduction about training and development of the diverse workforce, performance appraisal of a diverse workforce, job satisfaction, and diversity climate in a diverse workforce, some questions were raised such as, Do human resource practices have any connection to diversity climate? and Does job satisfaction have any role to play in shaping the connection between human resource practices and diverse climates? This research tried to address these questions by evaluating the degree of association between human resource practices and diversity climate along with an exploration of the connecting link of job satisfaction between these.

## Theoretical Framework and Hypotheses Development

As the number of diverse employees in the public sector grows, academics and practitioners in public administration are becoming more interested in the aspects of the employment structure that influence work-related consequences. The relationship between diversity programs and organizational success is particularly intriguing (Choi, 2011; Choi and Rainey, 2013; Walker et al., 2013; Opstrup and Villadsen, 2015; Sabharwal et al., 2016). Scholars believe a highly diverse workforce is damaging to productivity because of serious relationship conflicts caused by differences among employees, according to social categorization theory (SCT). In comparison, it has been proposed, based on information/decision-making theory, that diversity may be a human capital resource of diverse viewpoints that increases decision quality and performance (Pitts et al., 2010). Employees’ common opinions regarding a set of human resource procedures geared at identifying and embracing differences between individuals are referred to as diversity climate (Choi and Rainey, 2013). This diversity environment, in particular, includes shared organizational perceptions about how devoted a company is to diversity-friendly management practices (e.g., multicultural training, hiring, and mentorship) and diversity management to appreciate all demographic groups. According to optimal distinctiveness theory, the person possesses concurrent but opposing needs: belongingness and dissimilarity or uniqueness.

Individuals strive to attain the highest levels of their identities by balancing these requirements. In other words, whereas social categorization theory or information/decision making theory both emphasize the feeling of belonging and uniqueness, the optimal distinctive theory claims that individuals achieve an ideal amount of inclusion by balancing two competing requirements (Brewer, 1991; Hornsey and Jetten, 2004). This shows that whether the organization’s culture exhibits an appropriate commitment to diversity *via* the inclusion of all employees, regardless of socioeconomic category, is a vital aspect of successful diversity management (Shore et al., 2010; Johansen and Zhu, 2016). These theories provided the basis for the significance of diversity climate in workforce diversity management. Based on general systems theory, Hofkirchner and Schafranek (2011) and Jacobs (2014), different human resource practices are considered under a system of human resource management and the training and development along with performance appraisal of a diverse workforce are drawn in this study as contributing factors of managing the diversity climate. Blau established the social exchange theory, stating that when companies embrace human resource practices, workers feel that their company cares about employees (Blau, 1964), so they acquire favorable attitudes toward the jobs, which leads to job satisfaction (Allen et al., 2003).

Therefore, Andreassi emphasized the necessity for studies to clarify why human resource management techniques affect the level of job satisfaction in a multicultural setting (Karin Andreassi et al., 2014). Dartey-Baah and Ampofo (2016) stated that various people interpret satisfaction in different ways, but what one person considers acceptable may be considered unsatisfactory by another. Personal disparities in sense of achievement might be due to personal qualities or cultural variances. As a result, when firms deal with diverse workforces, evaluating job satisfaction and performance becomes more complicated. For example, Lim and Ling (2012) argues that when companies use the finest selection and recruitment methods, workers perceive that the company is dedicated to providing equality of opportunity, resulting in employee happiness. Furthermore, because the company recruits and selects the top applicants, these workers perform better in the workplace. Likewise, providing training and development opportunities makes employees feel like the company cares about their professional development, which leads to increased job satisfaction. Therefore, based on the social exchange theory, job satisfaction could mediate between training and development of a diverse workforce and workforce appraisal to diversity climate.

### Training and Development, Performance Appraisal, and Diversity Climate of Diverse Workforce

Kossek et al. (2006) noted that, while diversity training may cause short-term employee disagreement, the external trainer engaging in diversity training may assist in achieving greater productivity in the long run. Performance appraisal systems, employees’ job satisfaction, and job performance have all been the subject of much research in the past. Fair and transparent performance appraisal systems, according to previous research, assist firms in satisfying their employees, which leads to improved job performance. In this case, the source of conflict is that a traditional appraisal method may not be as effective or appropriate when working with such a diversified workforce. Companies should establish more customized performance appraisal methods that can meet the demands of multicultural workforces in a diverse setting. The performance evaluation committee, for example, should reflect all of the company’s cultures, ethnicities, and ages (Awais Bhatti et al., 2014; Aldoghan et al., 2019; Bhatti et al., 2019). In this context, Abidi et al. (2017) advised that minorities be involved in the committee that evaluates, selects, and promotes individuals in order to properly execute the performance assessment method in a diverse workforce.

Moreover, rather than focusing on personality, the assessment language should focus on performance. According to Yang and Hwang (2014) only a small amount of research has looked at the link between work happiness and job performance. Different individual components which affect job satisfaction could explain the conflicting results. While prior studies have also shown that job satisfaction seems to be an essential factor that impacts job performance, human resource professionals are still attempting to figure out what role job satisfaction plays in the success of multicultural workforces (Biswas and Varma, 2012). Biswas and Varma (2012) stated that globalization has significantly altered the type of work for workers, posing a tough opportunity for management seeking to improve worker job satisfaction. Workers develop these opinions based on organizational policies, processes, and workplace conditions, yet opinions about the value of workplace diversity might differ from one worker to the next. Workplace diversity policies and programs, according to Jin et al. (2017), play an essential role in improving the diversity climate.

The human resource department should take an active part in building an inclusive company atmosphere and effectively managing diversity concerns in this respect. Employee perceptions of diversity and prejudice in the workplace, according to Mor Barak et al. (1998), generate a diverse atmosphere. Madera et al. (2016) stated that previous studies had overlooked the importance of examining a good diverse atmosphere and work happiness. Furthermore, McKay et al. (2010) concluded that studies have begun to show the benefits of a positive diversity atmosphere, such as increased job satisfaction and organizational dedication. Moreover, Kim et al. (2015) argues that when employees believe that organizational policies and procedures apply equally to all workers irrespective of gender, language, color, or ethnicity, their organizational commitment and job satisfaction rise. Employees generally expect organizations to care about employees’ development and growth, and also provide a diverse environment that sends a message to employees that their company is unbiased and concerned about their wellbeing (Greening and Turban, 2000). As a result, it may be argued that workers are content with their jobs when firms create a psychological diverse atmosphere. When it comes to diversity management, people are more satisfied with their jobs when they believe their company values diversity and avoids prejudice. Likewise, when multicultural employees believe their employer is committed to eliminating prejudice, promoting diversity, and caring for their wellbeing, growth, and development, they are more content with their work, which leads to improved job performance (McKay et al., 2010). The arguments about training and development and performance appraisal systems of a diverse workforce suggested that these human resource practices could have an impact on job satisfaction and diversity climate, so we devised the following hypotheses.

***H***_1_**:** *Training and development of diverse workforce have an association with diversity climate*.

***H***_2_**:** *Performance appraisal of diverse workforce has an association with diversity climate*.

***H***_3_**:** *Training and Development of diverse workforce leads to job satisfaction*.

***H***_4_**:** *Performance appraisal of diverse workforce leads to job satisfaction*.

### Mediating Role of Job Satisfaction

Many elements that impact employee job satisfaction have been identified in previous studies, primarily since job satisfaction improves employee productivity. To put it another way, happy employees function better on the job. Few researchers claim that personality features have a key role in increasing worker satisfaction, which leads to improved job performance (Organ and Lingl, 1995; Rashid et al., 2016). Some highlighted how behavioral factors have an impact on not just job satisfaction but also work performance (Yang and Hwang, 2014). Collectivism, for example, indicates a person’s level of happiness, and someone with a high level of cooperativeness is accessible and sociable. As a result, people who are more pleasant tend to mingle with others more quickly. This satisfies their social demands, and they are content with their work. Moreover, it supports them in obtaining assistance with job responsibilities that will enable them to improve their job performance (Mount et al., 1998). People with an agreeable personality, on the other hand, are completely invested in their profession, are industrious, and are effective planners, which leads to higher benefits.

This leads to increased job satisfaction, which then in turn improves job performance. Furthermore, extroverts are warmer than introverts and invest extra time engaging in social activities, resulting in a positive workplace atmosphere (Judge et al., 2003; Schmidt et al., 2006). It leads to increased pleasure and direct engagement without interfering with others, which improves their performance even more. Furthermore, according to Connolly and Viswesvaran (2000), people who lack emotional stability are more likely to have conflicts with others, which leads to lower job satisfaction, and a lack of peer support leads to poor job performance. Employees that are emotionally secure can withstand pressure, which leads to contentment (Peltokorpi, 2008). Furthermore, the training programs assist them in improving their work performance. Workers are also happier and work harder to enhance job performance when they believe their performance will be evaluated using a prejudice-free and dependable approach.

According to a few researchers, employees are more satisfied once they reach their aims. If such a worker wants to earn intrinsic or extrinsic incentives, they will be satisfied when they are given them and will strive hard to maintain them, which will improve his or her job performance. As a result, human resource strategies not only gratify workers but also assist them in improving job performance (Ileana Petrescu and Simmons, 2008; Lim and Ling, 2012). Finally, in terms of a diversity climate, whenever workers recognize that their company values diversity and treats all employees fairly, they are happier with their work and are more motivated to enhance their efficiency. Workers’ perceptions of organizational practices that encourage a diversity climate make them think that if they put in the effort in work, they will be rewarded, which will boost job satisfaction and a diversity climate (Madera et al., 2016; Ashikali et al., 2020). Hence, it was concluded that job satisfaction could mediate the relationship of training and development, performance appraisal systems of a diverse workforce, and diversity climate in the form of following hypotheses.

***H***_5_**:** *Job satisfaction mediates the relationship between training and development and benefits of workforce diversity*.

***H***_6_**:** *Job satisfaction mediates the relationship between performance appraisal and benefits of workforce diversity*.

**FIGURE 1:**
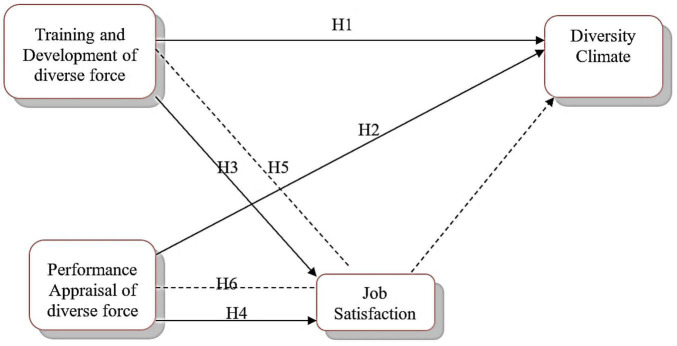
Theoretical framework.

The following conceptual model (Figure 1) has been formed based on the above literature and hypotheses.

## Methodology

In this quantitative study, the deductive approach has been used to test the hypotheses. The study follows a post-positivist approach since it checks the effects of effective human resource management practices, namely training and development, and performance appraisal on the diversity climate with the mediating role of job satisfaction. The questionnaire used in this study was designed on a five-point Likert scale ranging from strong disagreement to strong agreement. The participants were approached prior to the field study taking their consent for participating in the study. A self-administered survey was deployed to avoid the ambiguities and misunderstandings regarding the questionnaire. Data rationality was ensured by letting the participant fill questionnaires without any pressure. Further, the sample was selected on the basis of convenience sampling because it permits the researcher to obtain data in a cost effective manner and within relatively less time (Etikan et al., 2015). The target population of the study was comprised of the HR personnel in the HR departments of organizations in China. The sample size chosen for the study was 316. The unit of analysis for the present study was individuals working in HR departments in Chinese organizations.

### Statistical Tool

The statistical tool used for data analysis in the present study was Smart-PLS 3.3.3. The data was analyzed for structural equation modeling (SEM) with the help of this software. Furthermore, this software helps in developing the path models quickly, efficiently, and in detail (Dash and Paul, 2021). Using this software, the data is analyzed in two stages of measurement and structural model. The measurement model checks the validity of data with the help of factor loading, Fornell and Larcker Criteria, Heterotrait-Monotrait (HTMT) ratio, and average variance extracted (AVE). Moreover, the data reliability is checked through composite reliability and Cronbach’s alpha. The structural model generates the *p*-values, t-statistics, sample means, and standard deviation values which are used as criteria for acceptance or rejection of the hypotheses.

### Measurement

The scale used in this study to measure the independent, dependent, and mediating variables has been adapted from the previous studies that have then been further validated. The Cronbach’s alpha of each scale has been mentioned which is described to be above 0.6 for the acceptability of scale (Ismail et al., 2002). For example, the scale used for the variable training and development has been adapted from Bhatti et al. (2019) which consisted of ten items and showed the Cronbach’s alpha of 0.914 and thus falls under the acceptability criteria. Similarly, the scale of performance appraisal had also been adapted from Bhatti et al. (2019) which consisted of eight items having a Cronbach’s alpha of 0.906. Similarly, the variable diversity climate has been adapted from Mor Barak et al. (1998) consisting of four items with Cronbach’s alpha of 0.867. Furthermore, the mediating variable of the study, i.e., job satisfaction, has been adapted from Bhatti et al. (2019) comprising of three items with the Cronbach’s alpha of 0.873.

### Demographic Analysis

The results obtained related to the demography have been presented in Table 1. The demographic questions had been categorized into age, gender, education, and experience. The results show an equal participation for males (47.78%) and females (52.21%). Among the age categories, the highest number of participation was observed from age 31 to 40 while most of the respondents had master’s degrees. Most of the respondents had experience of more than 6 years, making them the most experienced, and had been actively involved in HR policymaking and implementation.

**TABLE 1:** Demographics analysis.

Demographics	Frequency	Percentage
**Gender**		
Male	151	47.78%
Female	165	52.21%
**Age (years)**		
20–30	118	37.34%
31–40	153	48.41%
41–50	39	12.34%
Above 50	6	1.9%
**Education**		
Bachelors	128	40.50%
Masters	164	51.90%
Ph.D. and others	24	7.60%
**Experience (years)**		
Less than 1	162	51.26%
1–3	88	27.84%
4–6	43	13.60%
More than 6	23	7.27%

*N = 316.*

## Data Analysis and Results

### Measurement Model

**FIGURE 2:**
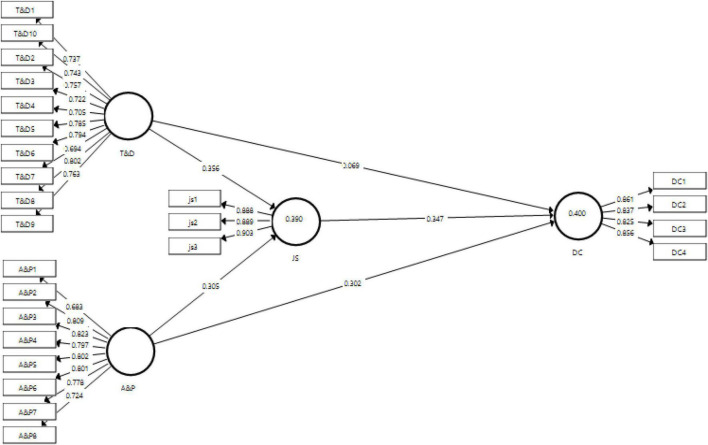
Output of measurement model. T&D, training and development, A&P, performance appraisal, JS, job satisfaction, DC, diversity climate.

The measurement model has been presented in Figure 2.

Table 2 explains the factor loadings, composite reliability, variance inflation factor, and the AVE for each variable. The minimum threshold for factor loadings usually practiced is 0.6 (Jordan and Spiess, 2019). All the values obtained as factor loadings for all variables are above 0.6, thus showing acceptability of the items. The minimum value obtained in the study for factor loadings is 0.683 for A&P1 which is also under the acceptable criteria. This table also shows the VIF which, according to Craney and Surles (2007) should be less than 5. The outer VIF values obtained in this study are all below 5, thus showing that no issues of collinearity are diagnosed in this study. The data was checked for AVE to identify the convergent validity of the scale. Literature suggests a value more than 0.5 should be observed for the variables in order to ensure the convergent validity (Rani et al., 2018). The values of AVE obtained in the study for all variables are above 0.5, having the lowest value of 0.564 for training and development. Moreover, the composite reliability obtained should be more than 0.7 (Chirumbolo et al., 2019). The reliability statistic obtained for each variable meet the criteria; hence, the reliability is present in the scale.

**TABLE 2:** Measurement model.

Variables	Factor loadings		VIF	Composite reliability	AVE
**Appraisal and performance**	A&P1	0.683	2.425		
	A&P2	0.809	2.424	**0.925**	**0.606**
	A&P3	0.823	2.579		
	A&P4	0.797	2.437		
	A&P5	0.802	2.478		
	A&P6	0.801	2.531		
	A&P7	0.778	2.298		
	A&P8	0.724	2.685		
**Training and development**	T&D1	0.737	2.224		
	T&D2	0.757	2.097	**0.928**	**0.564**
	T&D3	0.722	3.018		
	T&D4	0.705	2.510		
	T&D5	0.785	1.668		
	T&D6	0.794	2.952		
	T&D7	0.694	3.235		
	T&D8	0.802	2.164		
	T&D9	0.763	3.597		
	T&D10	0.743	2.312		
**Diversity climate**	DC1	0.861	2.110		
	DC2	0.837	2.058	**0.909**	**0.714**
	DC3	0.825	2.063		
	DC4	0.856	2.292		
**Job satisfaction**	JS1	0.888	2.315		
	JS2	0.889	2.323	**0.922**	**0.798**
	JS3	0.903	2.383		

*T&D, training and development; A&P, performance appraisal; JS, job satisfaction; DC, diversity climate; VIF, variance inflation factor; AVE, average variance extracted.*

Discriminant validity of the scales used in this study was measured using the tests of Fornell and Larcker criteria and HTMT (Heterotrait Monotrait) ratio to ensure that the variables are distinct from each other. The values in the HTMT ratio show discriminant validity if they are below 0.9 (Franke and Sarstedt, 2019), while for Fornell and Larcker criteria the topmost value in column should be highest among others (Henseler et al., 2015). Table 3 shows the results for HTMT ratio indicating the presence of discriminant validity as all the values obtained are below 0.9. Similarly, Table 4 shows the results of Fornell and Larcker criteria indicating the presence of discriminant validity with the placement of highest values at the top of their corresponding columns.

**TABLE 3:** Discriminant validity (HTMT ratio).

	A&P	DC	JS	T&D
**A&P**				
**DC**	0.627			
**JS**	0.653	0.642		
**T&D**	0.872	0.562	0.649	

*T&D, training and development; A&P, performance appraisal; JS, job satisfaction; DC, diversity climate.*

**TABLE 4:** Discriminant validity (Fornell and Larcker Criteria).

	A&P	DC	JS	T&D
**A&P**	0.778			
**DC**	0.559	0.845		
**JS**	0.584	0.564	0.893	
**T&D**	0.783	0.512	0.595	0.751

*T&D, training and development; A&P, performance appraisal; JS, job satisfaction; DC, diversity climate.*

R-square, F-square, SRMR, and NFI have been used to measure the goodness of fit for the current model (Elsayed and Aneis, 2021). The factor of determination (R-square) should be near to 1 for the higher fit to the regression line. In this study, the R-square value for the variable diversity climate is 40% while for the variable job satisfaction it is 39%, thus showing a relatively good fit. Furthermore, the F-square values are also used to check the model fit. According to Sarstedt and Cheah (2019), a value of equal to 0.02 shows poor fit, equal to 0.15 show fair fit, and above or equal to 0.35 shows good fit. In this study, highest F-square value obtained is for the relationship between training and development and job satisfaction which is 0.08, followed by 0.06 for the effect of performance appraisal on job satisfaction, and 0.056 for the effect of performance appraisal on diversity climate.

### Structural Model

**FIGURE 3:**
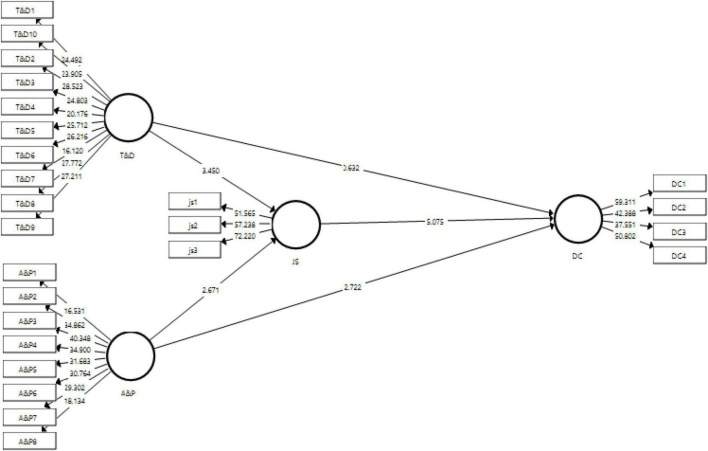
Output of structural model bootstrapping. T&D, training and development, A&P, Performance appraisal, JS, job satisfaction, DC, diversity climate.

The present study has used t-statistics, *p*-values, and original sample as the acceptance criteria for the hypotheses of the study. These values are generated by the structural model. The output for structural model has been shown in Figure 3.

The direct effects of the study have been presented in Table 5. The hypotheses are tested at 95% confidence interval, thus making the acceptance criteria at *p*-value less than 0.05 (Andrade, 2019). Furthermore, Table 5 presents the value of inner VIF that is also used to diagnose the collinearity issue, if any exists. For t-statistic to be significant, it should be more than 1.96 (Winship and Zhuo, 2020).

**TABLE 5:** Direct effects.

Paths	H	O	M	SD	Inner VIF	T-statistics	*P*-value	Results
T&D → DC	H_1_	0.069	0.077	0.108	2.797	0.632	0.527	Not Supported
PA→DC	H_2_	0.302	0.298	0.111	2.741	2.722	0.007*	***Supported***
T&D→JS	H_3_	0.356	0.359	0.103	2.589	3.450	0.001*	***Supported***
PA →JS	H_4_	0.305	0.305	0.114	2.589	2.671	0.008*	***Supported***

p* < 0.05, H, hypotheses; O, original sample; M, sample mean; SD, standard deviation; T&D, training and development; A&P, performance appraisal; JS, job satisfaction; DC, diversity climate.

The first direct effect indicates the first hypotheses (H1) of the study which is about the effect of training and development of a diverse force on the diversity climate. The *p*-value for this hypothesis is greater than 0.05, indicating no effect, and hence rejecting the first hypotheses. The H2 of the study is about the effect of performance appraisal of a diverse force on the diversity climate which shows the t-statistic = 2.722 and *p* < 0.05, thus supporting the second hypotheses. Training and development have shown to have a significant effect on the job satisfaction with t-statistic = 3.450 and *p* < 0.05, thus accepting H3. The last direct effect of the study is indicated by H4 that performance appraisal of a diverse force has an effect on job satisfaction and has been accepted with *t* = 3.450 and *p* < 0.05.

The mediating role of job satisfaction between performance appraisal and diversity climate has been indicated in the fifth hypotheses of the study. The results showed that job satisfaction plays a significant mediating role between the performance appraisal of a diverse force and diversity climate with t-statistic = 2.336 and *p* < 0.05 (see Table 6). Similarly, the second hypotheses of mediation has been indicated by H6, which has also been found significant with t-statistic = 2.711 and *p* < 0.05, thus supporting the hypotheses developed.

**TABLE 6:** Indirect effects.

Paths	H	O	M	SD	T-statistics	*P*-value	Results
T&D → JS → DC	H_5_	0.124	0.124	0.046	2.711	0.007*	***Supported***
A&P → JS → DC	H_6_	0.106	0.104	0.045	2.366	0.018*	***Supported***

p* < 0.05, H, hypotheses; O, original sample; M, sample mean; SD, standard deviation; T&D, training and development; A&P, performance appraisal; JS, job satisfaction; DC, diversity climate.

## Discussion

Businesses have challenges as a result of cultural variations among personnel, but if these differences are successfully managed, organizations may make greater use of them. Unless the services are dependent on feedback from the multicultural workforce, organizations can obtain a competitive edge. Furthermore, this can only occur if a multicultural workforce is given the chance to participate in the service and product creation process. Several researchers in the past have explored different human resource practices contributing toward the job performance of the employees in confined workforce setups. Some research has been carried out so far in terms of evaluating the impact of human resource practices on the job performance of a diversified workforce in diverse organizations. The education sector has been researched in the recent past, for example Awais Bhatti et al. (2014) in which the faculty members from diverse backgrounds were asked about the benefits and challenges of a diversified workforce. This research produced some very promising results indicating that job performance could be improved if the proper human resource management activities are performed in the organizations on a regular basis such as training and development of the diverse workforce along with good performance appraisal systems available in the organizations.

The current research evaluated the association between different human resource practices leading toward the promotion of a diverse climate as suggested by research (Awais Bhatti et al., 2014). Training and development of the diverse workforce was supposed to have a good impact on the diverse climate as suggested before, but our research indicated that training and development of a diverse workforce could directly affect the diversity climate and any sort of mediation was required which could lead to a better diversity climate. The outcomes of this study indicate that multicultural workforce job satisfaction influences the psychological diversity atmosphere both directly and indirectly. Similarly, Jin et al. (2017) noted that firms reassure as well as encourage a diverse atmosphere when they embrace strategies and tactics that treat all employees equally. Organizations that follow standard rules and processes that guarantee equal opportunities also increase employee happiness. Furthermore, workers agreed that their firm’s diversity or equal opportunities are available when they follow discrimination-free rules and processes. The results of the current study also showed accordance with Bhatti et al. (2019) showing that training and development could have an impact on job performance and diversity climate while direct relationship needed a mediator for its proper association with diversity climate. Furthermore, the other direct effects of workforce performance assessment on diversity climate and job satisfaction also proved that these human resource practices are needed in any diverse organizations for developing and positively prompting and impacting the diversity climate. This could lead to the improved job performance of the diverse workforce.

The indirect role of job satisfaction proved that the impact of certain human resource practices, like training and development of the diverse workforce and performance appraisal system of the organizations, is boosted through the mediation of job satisfaction for developing the diversity climate in the diverse organizations. This argument is supported by various pieces of previous research that evaluated the direct as well as mediating role of job satisfaction (Organ and Lingl, 1995; Connolly and Viswesvaran, 2000; Ileana Petrescu and Simmons, 2008; Rozkwitalska and Basinska, 2014; Yang and Hwang, 2014; Bhatti et al., 2019; Li and Xie, 2020). The results of the current study provide a novel basis for future researchers about utilizing some other human resource practices effectively in different diverse organizations for developing a sound diversity climate for employees.

### Practical Implications

The results of the study have indicated certain practical insinuations of the present study. First, the HR managers and the leadership teams of the diverse and multinational organizations should take the diversity climate and employee job satisfaction into consideration when devising their organizational policies in accordance with the diverse workforce. The present study also suggests multicultural organizations should incorporate the anti-discriminatory legislative clauses in their code of conduct to make a secure diversity climate among the employees. This feeling of ease would make the diverse employees feel higher satisfaction with their job and thus provide better overall performance. Furthermore, the training conducted in multinational corporations should consider the interpretation of training into different languages to make diverse employees feel accepted and valued. The employees from diverse workforces should be included in the appraisal process to encourage minorities and employees from dissimilar and diverse ethnicities to have an active participation in the organizational development.

### Theoretical Contribution

The present study has some vital theoretical input for the literature. First of all, the present study contributes to human resource management and organizational behavior by determining the effect of training and development on the diversity climate and the job satisfaction of the diverse workforce in multination organizations. In this research, the authors have revealed that training and development does not have a direct effect on diversity climate however it has been found to have an impact on the diversity climate through the job satisfaction of employees. Furthermore, it has also checked the role of another important human resource practice, performance appraisal, on job satisfaction and diversity climate. Results of the study have established evidence that performance appraisal plays an important role in the diversity climate of multinational organizations and that job satisfaction is a vital mediating variable in this process.

### Limitations and Future Recommendations

The first limitation of the study is that recruitment and selection and compensation and benefits have not been considered. It is important to check the effect of all HR practices on the job satisfaction and diversity climate to get more insightful results. Another limitation is that the present study has checked the mediating role of job satisfaction; however, more variables can be incorporated in the framework that can possibly mediate the relationship such as self-efficacy, leadership, etc. Furthermore, other moderating variables could also be added to enrich the framework such as emotional intelligence or organizational support. Therefore, future research should be carried out considering these suggestions.

## Conclusion

Effective HR practices are mandatory for the success of multinational organizations with diverse workforces. If not managed adequately, it could have many consequences in terms of a poor diversity climate among the diverse employees. In order to examine the effect of training and development and performance appraisal of diverse workforces, the current study has analyzed the impact of HR practices on the diverse climate with the mediating role of job satisfaction. The research study has found that, among HR practices, training and expansion does not have a significant impact on the diversity climate, however, performance appraisal had a significant impact. Furthermore, the mediating role of job satisfaction has been found to be promising mediator between HR practices and diversity climate.

## Data Availability Statement

The original contributions presented in the study are included in the article/supplementary material, further inquiries can be directed to the corresponding author.

## Ethics Statement

The studies involving human participants were reviewed and approved by the University of Science and Technology of China (USTC). The patients/participants provided their written informed consent to participate in this study. The study was conducted in accordance with the Declaration of Helsinki.

## Author Contributions

HM conceived, designed, wrote the manuscript, read, and agreed to the published version of the manuscript.

## Conflict of Interest

The author declares that the research was conducted in the absence of any commercial or financial relationships that could be construed as a potential conflict of interest.

## Publisher’s Note

All claims expressed in this article are solely those of the authors and do not necessarily represent those of their affiliated organizations, or those of the publisher, the editors and the reviewers. Any product that may be evaluated in this article, or claim that may be made by its manufacturer, is not guaranteed or endorsed by the publisher.
